# Factors influencing quality of transnational education: Evidence from a Sino-UK MA program

**DOI:** 10.3389/fpsyg.2022.1099359

**Published:** 2023-01-09

**Authors:** Changqing Zheng, Huhua Ouyang

**Affiliations:** Faculty of English Language and Culture, Guangdong University of Foreign Studies, Guangzhou, China

**Keywords:** service quality, student personal growth, internationalization of higher education, student satisfaction, sustainability of TNE

## Abstract

With the internationalization of higher education, transnational education (TNE) has gradually become one of the main means to mobility of education resources in many countries. There have been many studies on service quality research, but not many of these studies attempt to directly explore the in-depth factors contributing to service quality. This study investigated the factors impacting the quality of TNE based on the interpretation of students’ experiences on a Sino-UK MA program. Semi-structured interviews with seven participants revealed that high-quality educational resources and professional and sympathetic lecturers are the two major factors impacting the service quality. The results suggested that educational resources that meet the local social needs are considered desirable service offerings. Lecturers are key factors guaranteeing the service delivery. This study sheds light on service quality components of TNE that have not been given much attention in the literature of previous research, as well as informs host and export institutions on TNE programming.

## Introduction

With the trend of globalization, the international higher education sector has developed rapidly in the last four decades (Maringe et al., 2012), enabling educational resources to move around the world (Altbach and Knight, 2007; Rumbley and Wit, 2011). Studying abroad, as traditional internationalization activity, is gradually giving sway to a new landscape where diverse types of educational providers send their products to students’ own countries and establish campuses across borders (Cai et al., 2013). This kind of educational activity is termed Transnational Education (TNE) to describe “any teaching or learning activity in which the students are in a different country (the host country) to that in which the institution providing the education is based (the home country)” (Global Alliance for Transnational Education, 1997, p. 1). Education services that are provided to another country in accordance with host country’s regulations and contexts become the main threat to the quality around the world. Trifiro (2018) highlights the vicious circle of quality in TNE, in which the diversity of approaches and regulatory frameworks for TNE across the participating regions leads to a general lack of information and knowledge for other countries, and then results in a “trust gap” between foreign and host institutions, and finally causes the low level of collaboration in service quality. Such a trust gap is understandable, since the quality assurance conducted by export institutions depends to a large extent on the experience of their successful domestic programs, ignoring the context and needs of the host countries. In addition, “rogue providers” might exploit regulatory loopholes in the host countries by capitalizing on the prestige of renowned universities in their home countries. In fact, “rogue providers” are widespread especially in fast-growing markets such as Southeast Asia where demand for high quality education services exceeds supply of local institutions (Cremonini et al., 2012).

According to Harvey and Green (1993), the purpose of quality assurance in higher education is to gain better institutional performance to comply with external pressure, attract students, and maintain professional ethos. This study aims to provide quality assurance evidence to promote sustainable development in TNE by exploring the factors affecting the service quality. This study will inform host and export institutions on the improvement and maintenance of high-quality TNE.

## Literature review

The relevant literature is organized into three categories: first, transnational education, which reviews the landscape of TNE and addresses the quality assurance problems; second, service quality, which briefly reviews the development of service quality in higher education and introduces the dimensions of service quality; third, transformative learning theory, which introduces the framework to understand student personal growth in the TNE.

### Transnational education

The global Higher Education system is not uniform and homogeneous, and universities in different countries are characterized by their own performance standard, which in turn influences their student and staff mobility patterns. The normal direction of student and staff mobility is from poor, underdeveloped countries to the rich nations (Jones and de Wit, 2014; de Wit and Altbach, 2021). In addition, the global Higher Education system is characterized by increasing competition for students, resources, staff and funding (Maringe et al., 2012; Thondhlana et al., 2020). Therefore, universities are tending to keep their graduate attributes in line with globalization imperatives, fostering the internationalization attributes such as competences in intercultural communication, international literacy, international consciousness, global awareness and global perspectives (Maringe et al., 2012).

TNE, as one form of international education, providing cost-effective forms of international learning with limited or no overseas travel, has been rapidly developed over the years (Ilieva et al., 2017). But there is still an absence of systematic research in this area, partly due to disregard shown toward TNE as an area of Higher Education strategy and policy in the research agenda of Higher Education institutions and government bodies (Tsiligiris and Lawton, 2018). In the TNE landscape, local governments usually impose serious regulatory obstacles, including restrictions on foreign investment in education, workforce and migration restrictions and profit-repatriation controls (Tsiligiris et al., 2021). In addition, most host countries limit the type of foreign providers or the types of qualifications and accept only high-ranking universities or renowned qualifications that provide cooperation (Trifiro, 2019). Although host countries benefit from increased access to international higher education and improvement in education quality, and students gain skills in their international outlook and inter-cultural competencies (William, 2021), no evidence shows that TNE providers tailor the special program for host countries (British Council and DAAD, 2014). In practice, there is inter-cultural communication disagreement between institutions from Western developed countries and developing countries, because the former takes an egalitarian student-centered approach while the latter prefers hierarchical structures embracing control over students and the actions of faculty (Mirkasimov et al., 2021). Developing a share-culture in management and quality assurance is one of the challenges for the sustainability of TNE. Two main challenges for TNE risks are the assurance of quality and the maintenance of partner relationships (Healey, 2015). Some authors argue that trust and power asymmetries are key factors impacting on sustainable partnerships (e.g., Maselli et al., 2006; Bradley, 2007; Olsson, 2008; Kinser and Green, 2009). Others argue that there are tensions between commercial priorities and academic, which may lead to a risk of falling standards (Mcburnie, 2008), such as the increased opportunities for corruption of fraudulent diplomas and risks to academic integrity.

To sum up, challenges and opportunities coexist in the TNE landscape around the world. On one hand, TNE offers cost-effective opportunities for internationalization of Higher Education in developing countries. On the other hand, quality concerns have been stressed by host countries, which require a mutual understanding of TNE service quality by both collaborative institutions and countries.

### Service quality in higher education

Regarding quality, the first references appear in Aristotelian Greek philosophy. Later, Francis Bacon presents two forms of quality, namely, objective quality and subjective quality (Soares et al., 2017). From the 1980s onwards, with the development of the service sector in the global economy, management literature increasingly accepts the idea that objective quality connects to the product and subjective quality is associated with service (Grönroos, 1982; Zeithaml et al., 1988). Zeithaml (1998) further explains that quality of service is a construction of a complex nature and a result of the sum of the unique features applied to services, in the way they are perceived by individuals. Since the 1980s, quality assurance has become a standard instrument of higher education policy (Cremonini et al., 2012). Service quality is a vital concept in higher education as it provides the possibility for institutions to monitor the quality of their services and to seek improvements, but it is difficult to define the universal characteristics of this construct (Darawong and Sandmaung, 2019).

Service quality research in higher education is ever changing (Sultan and Wong, 2010), and there is significant debate about defining service quality in higher educational institutions (Becket and Brookes, 2006), since “it is a rather vague and controversial concept” (Cheng and Tam, 1997, p. 23). Over the years, there are two major approaches to understand the essence of service quality, namely, student-as-customer approach and student-deficit approach. Academics holding student-as-customer perspective suggest that service quality should be assessed by the perceptions of students (Aldridge and Rowley, 1998), since students are assumed to be the primary customers of the education service and they are also the product of the educational activities (Sirvanci, 1996; Senthilkumar and Arulraj, 2011). Because competition in higher education has become fierce (Hemsley-Brown and Oplatka, 2006; Curtis et al., 2009), institutions experienced pressure in capturing a bigger market share, which requires continuous improvement of service quality. As a result, institutions have been trying to gain competitive advantages over their competitors in the market, which enable the applicability of quality management principles, methodologies and tools from business context to higher education context (Khan et al., 2011). In contrast to the student-as-consumer approach, some scholars (e.g., Ploner, 2018) claim the “student deficit model,” in which international students are assumed sometimes to have unrealistic or even conflicting expectations of “service quality” and it is the students’ responsibility to adapt these expectations to “fit into” the institutions.

Student-as-customer approach originated from consumer marketing literature, in which satisfaction is “the consumer’s sense that consumption provides outcomes against a standard of pleasure versus displeasure” (Oliver, 1999, p. 34). In the context of education, research shows that students’ perceived service quality is an antecedent to student satisfaction (Guolla, 1999; Wilkins et al., 2012; Wilkins and Balakrishnan, 2013). As a result, Elliott and Shin (2002, p. 198) describe student satisfaction as “the favorability of a student’s subjective evaluation of the various outcomes and experiences associated with education. Student satisfaction is being shaped continually by repeated experiences in campus life.” And student satisfaction is demonstrated as an important indicator for evaluating service quality (Barnett, 2011). Samples could be found in recent studies in assessing student satisfaction and service quality in higher education (Weerasinghe and Fernando, 2017; Pedro et al., 2018; Yilmaz et al., 2018; Tijjani, 2019).

One of the major drawbacks of the student-as-customer approach is that it fails to identify the in-depth factors influencing the service quality, but only addresses the satisfactory or unsatisfactory items. Based on the items of original SERVPERF (Cronin and Taylor, 1992), Ahmad (2015) conducted a survey to six renowned international branch campuses operating in Malaysia. Ahmad employed a five-point Likert scale device to collect students’ perception data, and results showed that students were satisfied with the university reputation/image, program quality, lecturers and teaching quality, student learning environment, effective use of technology, counseling and academic advising support, and social life (direct/indirect) facilities. Based on SERVQUAL framework (Parasuraman et al., 1985, 1991), Zhong et al. (2012) conducted a quantitative survey to 1,800 students from 3 TNE institutions and 38 TNE programs in China. The questionnaire’s results showed that significant differences between the expectations and the importance of service quality have been found. Three satisfied dimensions in this study are service management, school-running conditions and affective engagement, in which the degree of service management satisfaction is the highest and the affective engagement is the lowest. Both large scale surveys above failed to explore the in-depth factors that influence service quality. Reasons are twofold. First, the Likert scale device with Importance–performance indicators employed by these studies aimed to identify the gap between expectations and performance about certain dimensions and was not designed to explore the factors that influence the quality. Second, quantitative data analysis emphasized descriptive results from students’ responses, ignoring social factors underpinning students’ attitudes and perceptions. Therefore, in this study, student satisfaction is seen as an important indicator of understanding the quality of service of the TNE program, while student personal growth is seen as another factor that serves to understand the quality of service.

Previous studies on student satisfaction contribute to the dimensions that relate to service quality in higher education. Based on Ahmad’s (2015) evaluation of Malaysia International Branch University, Zhong et al.’s (2012) satisfaction analysis of Chinese TNE and Liu et al.’s (2016) evaluation on instructional satisfaction for Chinese TNE, this study concentrates the service quality on the following ten dimensions: university reputation, program and curriculum design, lecturers and teaching quality, academic support, tangible facilities, service management, affective engagement, assessment and feedback, personal development and introducing foreign educational resources. Table 1 shows the dimensions and explanations for investigating the service quality in the current study.

**Table 1:** Dimensions of service quality.

Dimensions	Explanation
University reputation	The university is internationally known and recognized at home and abroad, and the degrees from the university are more prestigious. Graduating from university gives you a chance to get a better career and higher salary.
Tangible facilities	The university offers modern learning and teaching facilities, and the infrastructure is adequate. The library meets learning needs.
Program and curriculum design	The course of the program is made relevant to the context and intellectually stimulating. The program is accredited by international bodies and prepares students for their future career.
Lecturers and teaching quality	Lecturers are experts in the subject area and well prepared academically. The lecturers can stimulate students’ interest.
Academic support	Academic counseling service is always made available to the students. Students are able to find an official to advise on academic matters.
Assessment and feedback	The assessment criteria are clear and are made open to the students. The feedback is detailed and effective.
Introducing foreign educational resources	The number of foreign lecturers is adequate, and the introducing courses and textbooks are adapted for local students.
Service management	The faculty is willing to offer help to students and help to set up students’ security and confidence.
Affective engagement	The faculty understands students’ personalized needs and deals with students’ problems sincerely. The school pays attention to every student and supplies fast service.
Personal Development	The program offers help in students’ development of personal skills, in this study, the transformation.

^*^Dimensions were adapted from Ahmad (2015), Zhong et al. (2012), and Liu et al. (2016).

Dimensions in Table 1 inform the design of interview outlines, as well as the coding in data analysis. For example, the interview questions about making decision to join the program mainly focuses on the reputation and recognition dimension, and for interview questions about learning experience cover dimensions related to service delivery, such as teaching and academic support, introducing foreign educational resources, and service management. In the data analysis procedure, dimensions help to interpret students learning experiences, and generate the understanding of students’ sense-making. These dimensions are also discussed with the five-dimensions model introduced by Parasuraman et al. (1985) in the discussion section.

In summary, previous studies on service quality inform the philosophical assumptions and the dimensions to understand service quality in this study.

### Transformative learning theory

Transformative learning theory is one of the most intensively researched theories in the field of adult education (Taylor, 2005, 2008; Taylor and Snyder, 2012). The label transformation was first applied in Mezirow and Rose’s (1978b) study of the United States women’s resuming postsecondary study, which aims to “identify factors that characteristically impede or facilitate” the learners’ progress. In this study, transformative learning theory is employed to explore the factors impacting students’ change, which is considered the outcomes of education service.

Mezirow (2000) provides the 10 phases of personal transformation to identify factors and characteristically impede or facilitate the learners’ progress (see Table 2). According to Mezirow, learners’ transformation originates from the disorientating dilemma, and learners who experience the dilemma will initiate a critical reflection on themselves (phase 2) and the context (phase 3), which leads to a reflective communication (phase 4 and 5), and finally result in the action of transformation (phase 6–10).

**Table 2:** Ten phases of transformative learning Mezirow’s (2000).

Phase 1	A disorienting dilemma
Phase 2	A self-examination with feelings of guilt or shame
Phase 3	A critical assessment of epistemic, sociocultural, or psychic assumptions
Phase 4	Recognition that one’s discontent and the process of transformation are shared and that others have negotiated a similar change
Phase 5	Exploration of options for new roles, relationships, and actions
Phase 6	Planning a course of action
Phase 7	Acquisition of knowledge and skills for implementing one’s plans
Phase 8	Provisional trying of new roles
Phase 9	Building competence and self-confidence in new roles and relationships
Phase 10	A reintegration into one’s life on the basis of conditions dictated by one’s perspective

Research on transformative learning suggests that certain conditions lead to transformation. Liu (2013) points out that transformative learning conditions could be categorized into internal and external conditions, in which the internal conditions include learners’ characteristics like age, previous experience, interest, motivation, metacognitive competence and learning strategies, etc., and the external conditions include factors facilitating learning like context, teacher and learning content, etc. The transformative learning theory was employed to understand the internal and external factors that influence the service outcome, here the learners’ personal growth.

## Research method

This qualitative case study used a purposive sample to explore factors influencing TNE service quality. Themes were developed from convergence of three data sources: semi-structure interviews, program documents and student evaluation feedback forms. Data were inductively analyzed, and themes were generated to provide an in-depth description of the case of TNE program.

### Data collection procedures

The sampling case of this study is a Sino-UK MA TESOL program, which has been running since 2003, as one of the pioneers of transnational activities in China, after the release of first formal regulations on transnational educational in China. This program is joint by University of L (hereinafter refers “L University”) from United Kingdom and University of G (hereinafter refers “G University”) in China. The program aims at training TESOL lecturers and teachers working at tertiary, secondary or primary schools in Chinese education system. All teaching takes place at the G University premises, and L University sends staff to deliver face-to-face teaching. The program lasts one academic year, just like other full-time MA programs offered at L University, from September/October to August/September of the following year. The program enrolls up to 30 students each year, and the entry requirements are the same as those for the L University-based MA TESOL program. Those who are accepted onto the program need to have to meet the following requirements: (1) a first degree in English, from a Tier 1 or 2 university (the Tier 1 and 2 university mainly includes state-running universities and colleges in China), with GPA of at least 75%; (2) a teaching qualification; and (3) normally a minimum of 2 years post-qualification TESOL experience.

I have been working on this program for over 10 years, as an educational service officer. With repeated interactions with students, I have the chance to know the students well, helping me to identify the most informative participants. The target participants are graduate students, since they have experienced the whole learning process and become familiar with the service provided by the program. Furthermore, after finishing the program, students normally have some reflection on their study, which provides an insight into their changes. Out of convenient reasons, I sent invitations to the graduates with strong connections who knew I was doing service quality research on this program. Most of them were interested in participating in this research.

Seven participants were invited to the current study, their demographic information was stated in Table 3. They are female, experienced EFL teachers, having graduated from this program. All participants used pseudonyms from an alphabet order from A to G.

**Table 3:** Participant demographic information.

Participant ID	Name	Gender	Age	Entry year	Teaching context
Participant 1	Alice	Female	29	2020	Test preparation training class
Participant 2	Betty	Female	30	2020	Primary school
Participant 3	Cindy	Female	34	2015	Small size training class
Participant 4	Daisy	Female	35	2020	Test preparation training class
Participant 5	Emma	Female	43	2020	EFL teacher educator
Participant 6	Flora	Female	35	2017	International school
Participant 7	Gloria	Female	31	2020	Secondary school

One-to-one semi-structured interviews were conducted with each participant with the purpose of exploring their perceptions of the service and their reflections on personal growth. Each interview approximately took 45–60 min and was recorded with the interviewee’s permission. Based on the research question, the design of interviews questions was divided into two parts. The interview questions in the first part are in chronological order according to the program schedule with the purpose of recalling participants’ memories easily. The questions in the second part are about the overall sense-making of the program. The interview questions are informed by the program evaluation strategy and cover the main student satisfaction dimensions and transformative learning phrases. The interview questions are used in a flexible manner to move from general issues to more particular issues. For example, interview questions in part one cover participants’ whole learning process in the TNE program, including before joining the program, studying in and graduating from the program. The questions are organized in a chronological order and focus on discovering participants’ interesting and important things. During the interview, I usually listen to the participants attentively and probe at certain points (e.g., when talking about the most impressive things during classroom teaching, I may ask about “Can you tell me the difference between Chinese and foreign lecturers in classroom teaching?”). Interview questions in part two mainly concentrate on the confirmation of learning experiences that participants described in part one. These questions are more abstract and related to the personal judgment of service quality.

The documentation data in the current research includes the Handbook of the MA TESOL Program and student evaluation feedback forms of one module. Documents analysis is used to triangulate the data for a comprehensive understanding of the service quality. The handbook provides basic information about the program operation, describing the curriculum design, the outcome and transferable skills, as well as the program structure and teaching arrangements. The documentation of student evaluation form is a sample of the student evaluation for the module delivery. A module delivered by lecturers from G University was selected as the sample, which contains feedback from 25 students in cohort 2019. This evaluation form was selected as the sample since it provides evidence to better understand the Chinese lecturer’s work in localizing the program to Chinese context. The feedback form contains three main sections. Section A and B employ a five-points Likert scale device to evaluate student satisfaction with the module content, lecturers, learning resources and other factors related to learning. Section C contains four open-ended comments on the modules, including the best aspects of the modules, the aspects that benefit further development, the realistic improvements to the module, and other comments on the module. The documentation of student evaluation feedback forms was used to provide triangulated evidence of satisfaction and personal development from the study of specific module.

The ethical issues were carefully considered in the current study. Ethical approval has been obtained from the ethics committee at the researcher’s institution before data collection. All students invited in the current study participated voluntarily with consent confirmation. All participants were required to read an information sheet and sign the consent form. They were well informed about confidentiality and the risk of attending the interview. In addition, participants were well informed about data ownership.

### Data analysis

The aim of data analysis in this study is to rigorously and creatively organize, find patterns in and elicit themes from data (Burnard and Morrison, 1994). There were only a few researchers providing inherently intuitive analysis procedures in case study (Tesch, 1990). In case study, there are two types of analysis: “within-case” and “cross-case” (Stake, 2006; Creswell, 2007). Cross-case analysis identifies the similarities and differences between themes across the case (Eisenhardt, 1989). The current study employs cross-case analysis to analyze themes across different participants’ accounts, in which different participants are viewed as different cases. The cross-case analysis helps to provide of the similarities and differences of the essence of the service quality in the TNE program. In this study, the data analysis first focuses on participants’ personal sense-making with the learning experience in the MA TESOL program and then evaluates the service quality in connection with the documentary analysis, with the commitment to an understanding of TNE program. The data analytic cycle in this study is iterative and inductive, and the analytic result is a co-construction of participants and researchers.

As I reviewed in the literature section, the service quality and transformative learning theory comprise the theoretical framework of this study, which informed the data collection and guided the data analysis. Specifically, the dimensions of service quality in TNE and the phases of transformative learning formulated the framework to understand students’ perceptions of service delivery and students’ personal growth, that is, the service outcome.

All interviews were transcribed into texts by machine voice recognition device and my manual editing. To avoid the inaccurate interpretation, interviews were transcribed and analyzed in the original language, Mandarin Chinese, and the English used for quotes in this paper were my translation of original texts. Each interview transcript as well as the audio recordings were imported into Nvivo 12.0 as a single file for data management and further analysis. The interview texts are analyzed under a constructivism paradigm with four major stages in data analysis, including: transcribing and reading, generating initial coding, developing emergent themes and writing up the themes. These four stages are consistent with Morse’s (1994) proposed data analysis framework: understanding, synthesis, theorizing and recontextualization.

In this study, the interview texts were coded with two rounds of noting. The first round of noting uses a repeated reading strategy to ensure that participants are the focus of analysis and avoid over summarization of complex information. At this stage, the noting work mainly focused on participants’ concerns, language and abstract concepts, under the framework of perceptions of service delivery, based on the satisfaction dimensions, and the perceptions of personal growth, based on the phases of transformative learning. The second round of noting based on the first round noting and my own interpretation, which was categorized into two classes, namely service delivery and transformations. The characteristics category contains tentative summary of emerging patterns about factors influencing students’ satisfaction of TNE quality, such as curriculum, teaching, facility and social life. The transformation category contains tentative summary of emerging patterns about students’ personal growth, such as dilemma, reflection and action. And then, documentation analyses were used to triangulate the interview texts by examining the program settings like objectives and teaching arrangement, as well as students’ evaluation on module delivery like best aspects of the module. Table 4 presents an example of codes and categories.

**Table 4:** Example of codes and categories.

Codes/free nodes	Categories	Tentative summary
1. Lecturers and teaching quality	Service delivery	1. Experienced one-year hard time a. Intensive lecturing b. Heavy written assignment c. …
2. Program and curriculum design		2. Foreign teachers are kind a. Necessary support b. Affective engagement c. …
3. Academic support		
4. …		
1. Dilemma	Transformation	1. Transformation of identity a. from teacher to practitioner b. from knowledge receiver to constructor c. …
2. Community communication		2. Transformation of perspective a. Being critical b. …
3. Reflection		
4. ….		

After the categorization of massive interview data, major themes emerged from these data by building links among different cases and triangulating the subthemes with documentation. The themes and codes were later reviewed by the second author, and the disagreements of coding were resolved through discussion. Finally, we reached an agreement on two themes and seven sub-themes concerning factors influencing the service quality. Table 5 presents the samples of generating themes from data sources in this study. In this example, different participants repeatedly mentioned the lack of EFL theoretical knowledge. The phenomenon could be traced back to the setting of “Chinese Undergraduate Specialty Catalogue of Higher Institutions” (released in 1989), in which “EFL teaching” was not given a specialty status. Therefore, most EFL teachers were the undergraduate degree holders of English or Education, and most of them lack theoretical knowledge. Combining data from program handbook and evaluation feedback form, the curriculum satisfied local needs could be found through the transformative learning framework.

**Table 5:** Example of generating themes and sub-themes.

Theme	1. High-quality Educational Resources
**Sub-theme**	**1.1. Curriculum based on local needs**
Data request	a. Interpretation of interview texts: Participants showed that this program offered theoretical knowledge of EFL teaching, which served as the supplement knowledge for their professional development. …I think the four modules are very helpful. It is a professional, systematic and complete system (Flora).“Before I joined the program, I encountered a bottleneck in teaching… with the continuous deepening of learning, we know that English teaching is difficult, or second language teaching is exceedingly difficult. Because it crosses ethnic and cultural backgrounds…” (Cindy).
	b. Program Handbook: the learning outcome requires the MA TESOL graduates to be able “to demonstrate in-depth, specialist knowledge and mastery of techniques relevant to the discipline…”
	c. Evaluation feedback: This lecturer can make abstract points clear and easy to understand; 2. Its context is practical and relevant to teaching (Extract 20) Pay much attention to teaching reality (Extract 21)

## Findings

### High-quality educational resources made relevant To host Country’s needs

The term “educational resources” was a broad concept, normally including tangible and intangible educational resources. This program is delivered in a Chinese university where most facilities were shared between students in this program and other local programs of G University. Due to the cross-border nature of TNE, the foreign educational resources are mainly intangible, such as curriculum and flying faculties. From the interview data, the intangible educational resources on the program are the major factor influencing the service quality. Four aspects of intangible educational resources emerged during the data analysis: curriculum, assessment, learning community, and institution reputation.

#### Curriculum based on local needs

The closed relevance of curriculum and needs of local society is the primary factor influencing TNE service quality. The reasons are twofold. First, there was a need of the theoretical and practical knowledge in EFL teaching in China. Second, there was a need of weekend program for in-service teachers. Evidence could be found from the extract of interviewees’ accounts as follows:

I think this program should be able to promote my future development (Alice).

When my front-line practical experience was rich… I may need improvement, that is, some theoretical improvement (Emma).

…I think the four modules are very helpful. It is a professional, systematic and complete system (Flora).

Before I entered the program, I felt that I had strong practical experience, but lacked theory, which made others feel that I am not professional enough, and now I can do this (Gloria).

“Before I joined the programme, I encountered a bottleneck in teaching… with the continuous deepening of learning, we know that English teaching is difficult, or second language teaching is exceedingly difficult. Because it crosses ethnic and cultural backgrounds, it is completely different from the basis of language” (Cindy).

Accounts from participants showed that this program offered theoretical knowledge of EFL (English as Foreign Language) teaching, which served as the supplement knowledge for their professional development. The phenomenon that EFL teachers lack theoretical knowledge in EFL teaching could be traced back to the setting of “*the Undergraduate Specialty Catalogue of Higher Institutions*” (released in 1989) in China, in which “EFL teaching” was not given a specialty status. Therefore, most EFL teachers were the undergraduate degree holders of English or Education, who normally feel the needs of theoretical knowledge in EFL teaching. The theoretical knowledge in EFL teaching provided by the curriculum was perceived as an important educational resource imported from foreign institutions. As stated in the handbook of the program, one of the learning outcomes is to describe and evaluate major approaches to the teaching and learning of TESOL and to demonstrate in-depth, specialist knowledge and mastery of techniques relevant to the discipline.

Additionally, the course content made localized to the teaching context in host country was perceived as another factor contributing to the quality, which could be captured from participants’ accounts as follows:

…Chinese teachers may know more about China, at least about our classmates, learning habits and other specific problems, so they will (pause), er, better meet your learning needs (Daisy).

Therefore, compared with foreign teachers, I think Chinese teachers’ teaching is based on our understanding, that is, they know the level of our understanding, so their explanation will be more systematic (Gloria).

Daisy and Gloria valued the teaching content delivered by Chinese due to the close connection to local teaching context. Apart from the teaching content, the teaching schedule of this program meets in-service EFL teachers’ needs. As the program structure stated in the program handbook, this program offered face-to-face teaching on Friday night to Sunday from 2017. The new arrangement offered students the rare chance to be possible to gain an MA degree without quitting the job.

With the support of curriculum, the learning outcomes of the program could be interpreted as learners’ transformation of identity under the transformative learning theory. The cross-case analysis suggests that students changed from teacher to practitioner after the study, which could be evidenced from Gloria and Flora’s account as follows:

I think the biggest change is the change in my understanding of my own positioning. Before, I thought I was a state-owned school’s teacher. I can foresee my retirement in 30 years. But now I find that I am no longer a language teacher only. Now I think I should position myself as a teacher who keeps learning and use such a learning attitude and spirit to influence my students. Although I think the end of my career is retirement, I can do a lot of different things in this process. I can test different teaching methods and new ideas in my students in my teaching. yes. Therefore, my positioning should be different, and my requirements will be higher… (Gloria).

…but after I graduated, under some theoretical guidance, I thought about why some students had special psychological problems and how I could go on to the next step to solve their problems. I used some theoretical knowledge to make my teaching more meaningful or comprehensive… (Flora).

According to Mezirow’s ten phases of transformative learning (Mezirow, 2000), the change that happened to Gloria and Flora can be understood as transformation since they were taking action as the new role. They were able to think about the reasons for classroom problems and attempted to improve through practice, which presented themselves as the reflective practitioner.

#### Reliable assessment and feedback ensuring academic success

Participants provided evidence indicating the importance of assessment and feedback to service quality by providing understandings of academic regulations and grading criteria. Evidence can be captured in the extracts as follows:

…students may still have some plagiarism problems because of their carelessness in the actual process of academic writing (Alice).

… (The details) like manner of quotation and format of citation were not specifically introduced, although we knew the importance of academic integrity. To avoid plagiarism, classmates need to be quite careful to check, and some mistakes were made by carelessness, but were identified as plagiarism… (Alice).

Alice highlighted the significance of assessment by complaining that the regulations of plagiarism were not clear enough. Another aspect concerning assessment is the grading criteria between Chinese and United Kingdom supervisors. In this case, students believed the foreign institutions hold the stronger power in grading decision making and might be stricter in marking, which could be captured as follows:

…we had always been concerned about the marker of dissertation. We believed that teachers from both sides do the marking together. But when I read the feedback of my dissertation, it seems that there was only one UK teacher… In other aspects, I felt that the UK side is stronger (in marking) (Daisy).

…some young classmates may feel that some supervisors seem more serious and demanding. They were afraid those supervisors would give low marks… (Emma).

Apart from the assessment criteria, the details of feedback played an important role in students’ perceptions of high-quality educational resources. Generally, students in this program showed gratitude for the feedback on their assignment, from which they improved their academic performance. The following extracts from Flora’s accounts provided evidence of the effectiveness of the feedback:

At that time, I was reading literature to get theoretical support, and then copied the theories from the literature to my paper. Then Mr. M (foreign tutor) gave me feedback about reading the literature, I should think about what is relevant to my practical teaching, how it supports my teaching, or what thoughts I get from the process of practice. It must be combined with my own practice, rather than listing a theory on the paper alone (Flora).

#### Learning community supports personal growth

According to the satisfaction dimensions in the literature review, the learning community can be categorized into social life dimensions. Social life dimensions in the literature emphasize tangible facilities, such as cafeterias, resting areas, accommodation and other leisure activities provided by the university. This subtheme borrows the terms “community of practice” from Wenger (2010) to describe the phenomena that students interacted regularly in resolving an issue, improving skills, and learning from others, which could be captured as follows:

The discussion is also quite frequent in social media, and the help is of significance (Alice).

I think we had a strong ability to search for resources. Up to now, our group is still highly active in sharing information (Daisy).

Both Alice and Daisy valued the sharing of literature among classmates, which is in line with the important function of the community, that is, resources sharing. Apart from this, participants in the learning group were inspired by other members of the community, as follows:

…I set up an online group with several classmates. In fact, all of us may have confusion in research sometimes. When I was confused, my classmates helped me change my thinking in time. They may have more insightful thinking. They did the thinking from the perspective of a tutor, and they acted as an observer and helped me do this (Daisy).

Daisy recognized that some classmates were more capable, so she looked for help in the community. The fact that she was inspired by classmates indicates the help of learning community, which could be echoed by Flora’s account as follows:

Then, in the classroom, the teachers and some students are very active and powerful. They have also made some achievements in the academic field. I think I have learned a lot from my classmates. (Flora)

The learning community plays an important role in students’ satisfaction of educational resources. Although the learning community was not provided by the institutions directly as the educational resources, it could be seen as the side effects of the program offerings since the high admission standard guaranteed peer’s academic competence in this program.

#### Institution reputation

The reputation of both institutions was one of the most important factors contributing to students’ overall satisfaction since it guarantees the competitiveness of the degree in the job market and the peers’ recognition, which could be captured from several participants as follows:

… I chose this program for many reasons. Of course, there must be a reputation for this program and the endorsement from L University and G University. (Betty)

the MA TESOL degree is shown to others that I graduated from a prestigious university. To a certain extent, the value of business will be higher. (Daisy)

In fact, I can get the experience of two universities in disguise through this program, from both L University and G University, and this (experience) gives me great confidence… (Emma)

The accounts stated above conveyed the similar idea that students valued the reputation of both institutions as an important resource. The prestigious name of both universities was considered as an important indicator for students who cared about the competitiveness of the degree. Furthermore, peers’ recognition of the program was considered as the crucial indicator contributing to reputation. Third, the admission requirements increase the reputation of the program. As stated in the program handbook, the minimum requirement of language proficiency in the admission requirement was fairly high.

### Professional and sympathetic lecturers offered supports to students’ success

This theme reveals that the lecturers in the program are the key factors contributing to service quality. The professionalism, affective engagement, responsiveness of the lecturers was perceived as important characteristics to deliver high-quality service.

#### Professionalism

In the interview, students perceived the professionalism of lecturers from their professional image, classroom teaching and effective academic support. The most convincing example of the lecturer’s professional image came from Betty, when she responded to the questions of the most impressive thing in the program, as follows:

I remember Dr. W was the first teacher I met on this program… he always emphasizes misconduct in academic research. So, I remember this word very clearly. I was influenced by his ideas from different angles (Betty).

The first perceptions of the lecturer were quite important. Alice, showed a similar perception about the first image, which could be captured in her account as follows, “… the most impressive thing was I found that the lecturer who teaches in the classroom is also the researcher of this area after class…” The reason for Alice perceiving the lecturer’s professionalism in research may be because she read the lecturers’ publications after class. In addition to the general image of professionalism, students had experienced professional training in the classroom, which contributed to the satisfaction of teaching quality. Gloria, expressed her gratitude to both Chinese and foreign lecturers, in which the Chinese lecturers were able to connect the context and theories in China, while foreign lecturers were good at inspiring students, as follows:

In terms of teaching, I feel Chinese teachers are very close to our reality, because some of the theories they talked about are things we really encountered in our teaching experience. Then I would feel that foreign teachers gave me a new perspective of thinking and research direction… (Gloria).

Gloria valued the classroom teaching since both Chinese and foreign lecturers conveyed professional knowledge from their own strengths. In other words, Gloria was satisfied with the quality of teaching because of the perception of professionalism on both sides. In addition to the lecturer’s professional image and professional classroom teaching, students perceived professionalism from lecturer’s academic support, which could be found at Floria’s response to the academic support and related questions as follows:

I felt that the UK teachers encouraged us to think and explore more. For example, they strictly required us to write a draft and an outline before a due date, or you can write as much as possible, and then they gave us very detailed feedback, including grammar and sentence suggestions… (Flora).

#### Affective engagement

From the interview, the lecturers’ affective engagement in meeting students’ reasonable demand, empathizing the defective students, and paying attention to students’ personal needs were perceived as high-quality service. Gloria provided an anecdote that how the lecturer changed the schedule to meet students’ reasonable demand as follows:

… because there was a traffic jam when we came home after class at 4:30 or 5:00. We could discuss it with the teacher and start at 2:00. The teacher was also willing to adjust it. But the UK teachers were very principled about the time, and it was not easy for us to discuss with them and reset the time… (Gloria)

Gloria compared the demand of the class time adjustment between Chinese and foreign teachers, and she showed gratitude to Chinese teacher who was willing to adjust. This small change showed the lecturer’s willingness to deal with students’ problems sincerely, from which students made sense of satisfaction and respect.

Empathy to defective students was perceived as another factor to satisfaction with lecturers. The term “defective students” here refers to those students who engage in a difficult position of academic success. Flora shared her experiences of being kindly treated by lecturers when she encountered the penalty of plagiarism:

But what makes me feel very warm is my two mentors, M and J. They participated in my meeting with the Academic Committee, encouraged me and impressed me. M encouraged me and said that this is a normal process to develop, because I have to take into account my work, and the time is very tight, and it is stressful. I felt that instead of being criticized, I was encouraged… (Flora).

The extract from Flora indicated that the lecturers’ empathy was important to students’ satisfaction. In addition, paying attention to students’ personal needs was perceived as another affection factor contributing to students’ satisfaction. Emma described her perceptions of meeting personal needs in classroom teaching as follows:

We have 30 teachers with different backgrounds in the program. When Dr. W talks to different people, he did it according to the student’s personal cognitive level or cognitive structure, because in terms of his academic competence, he could know very well what the student needs and help them by delivering what he needs (Emma).

Emma was a teacher educator with a deep understanding of teachers’ personal needs from the professional development perspective, and what she made sense of the classroom teaching presented the importance of meeting students’ personal needs in teaching quality.

#### Responsiveness

This subtheme was brought to the fore during the data analysis process because of the different expectations for Chinese and foreign teachers in responsiveness emerged. This phenomenon is worthy of special attention since it reveals the essence of students’ satisfaction with service in TNE, since the service was delivered by staff from institutions domestic and abroad.

One question regarding the responsiveness was added in the semi-structure interview, which included two sub questions: (1) Did you experience that foreign teachers do not reply to your email while they are on holiday? (2) Do you think it is acceptable if Chinese teachers do not reply to your email while they are on holiday?

All participants showed their understanding that foreign teachers do not reply to email on holiday. But when talking about Chinese teachers, the response varied. Some participants thought it was still acceptable, while some thought it depended. Daisy stated that it was not acceptable.

When Alice was asked about whether it is acceptable for Chinese teachers do not reply to your email while they are on holiday. She was surprised to hear the question and repeated the questions again. It seems that she never thought about this question before. She replied, “Although I have not encountered it, it is understandable that it depends on the urgency of my problem.” Alice showed her expectations that Chinese teachers should response to students’ questions on time. Daisy has made it clear that it was unacceptable for Chinese teachers not replied to email when they were on holiday, as follows:

Because it was a cultural reason. Because there were foreigners in my working environment before, I knew their working culture very well. I know that if they are on holiday, they are completely enjoying their holidays, unlike us, because we might be disturbed on holiday… (Daisy).

Although students showed different expectations on responsiveness between Chinese and foreign teachers due to the different working cultures, the lecturers’ prompt response to students’ needs was perceived as the important factor contributing to the service quality.

## Discussions

Transnational education was treated as a trade service (Nix, 2009), and students are considered as consumers of the educational service in this study. Findings from the Sino-UK MA program suggest the features of a high-quality service with some key components. These components of high-quality service and the sustainability of high-quality service are worth further discussions.

### Trichotomy of TNE quality factors

It is necessary to discuss what forms the high-quality service based on this Sino-UK MA program. Findings suggest that the curriculum, the assessment and feedback, the learning community and the institution’s reputation. These factors were mainly generated from student-center evaluation data. In fact, from a broader point of view, these factors above could be intertwined with the lecturer’s factor depicting a deep structure of high-quality educational resources. Some intertwined features could be summarized as follows. First, the program offers plenty of theoretical and practical knowledge, and the knowledge was originally delivered by professional lecturers who ensure the quality of teaching. Second, the assessment and feedback were conducted by the lecturers, and they ensure the reliability of the service. Third, special support was provided by the learning community and the sympathetic lecturers, in which the lecturers’ affective engagement and responsiveness were perceived as the important factor contributing to the effectiveness of service. From the discussion above, high quality can be categorized into three dimensions: service offers, service delivery and service outcomes as stated in Table 6.

**Table 6:** Dimensions of high-quality education.

Dimensions	Elements	Features
Service offers	Curriculum	Curriculum that provides theoretical and practical knowledge to satisfy local students’ needs in future development
	Assessment and feedback	Reliable assessment and feedback that ensures the academic standards and students’ progress
	Institution reputation	The reputation of the institution and the degree awarded that benefits students’ future career development or the competitiveness in the labor market
	Program structure	Well-designed structure that meets local needs and offers chances for students to experience success that has positive influence on self-efficacy
Service delivery	Teaching quality	Professional lecturers with expertise in the academic area that ensures the delivery of inspiring teaching and academic standards
	Responsiveness	Prompt and effective response to students’ needs or enquiries
	Learning support	Effective interactions with peers to solve the problem and affective assistance from the tutors
	Affective engagement	Staff taking actions to students’ reasonable demand, empathizing the defective students, paying attention to students’ personal needs
Service outcomes	Personal development	The program offers help in students’ development of personal skills
	Social development	Program cultivates students to better serve the host country’s social development

Mezirow (2000), adapted from Kitchenham (2008).

This trichotomy contributes a new framework to understand and evaluate the service quality of TNE program. Compared to the dimensions of service quality in previous studies borrowed from management literature, the new framework is well-structured and easy to monitor the TNE service quality, in which the service quality is combined with sociocultural factors in host countries. First and the most, the needs of local society and local students’ personal development is the primary factor to evaluate a TNE program. Second, the reliability of service delivery is an important safeguard measure. Third, the service outcome is an important means to verify education services.

### Mutual understanding and equal dialogue as the foundation of sustainable development of high-quality TNE

As discussed in the literature, trust and power asymmetries are key factors impacting on sustainable partnerships, and tensions between the commercial priorities and academic standards become another factor threatening the sustainability of TNE, since TNE increased the opportunities for corruption of fraudulent diplomas and risks to academic integrity. Therefore, a mutual understanding of quality assurance from both host and export institutions is one of the key factors to ensure the sustainability of TNE development. This Sino-UK MA program has been running for nearly 20 years and is one of the longest TNE programs in the world. Based on the study of service quality factors, a deeper conclusion could be summed up, i.e., mutual understanding and equal dialogue between host and export institutions is the basis of sustainable development.

The findings suggest that the mutual understanding of the needs of local society and students is the key factor for the success of this long-term TNE program. Mutual understanding is mainly reflected in two aspects. First, the mutual understanding of local needs. In this case, the lack of systematic knowledge in EFL teaching in China is the basis for cooperation in this MA TESOL program, which can be evidenced from participants’ accounts of their urgent need for training in theoretical and practical knowledge of EFL teaching. Based on such understanding, the foreign institution offers particular educational resources, and the local institution localizes the resources to local context. The localization of foreign educational resources is based on the understanding of local EFL teaching context and students’ characteristics that were mentioned in participants’ interviews repeatedly.

In addition, equal dialogue is the pathway for sustainable partnership. This Sino-UK MA program is designed to award foreign degrees only, which brings the inbuilt power imbalance in decision-making, which has been approved by the other joint program in the world (Knight, 2014, 2016). Findings in this case show the pattern of an equal dialogue between host and export institution in programming, which can be summarized in two levels, namely, the dialogue between institutions and the dialogue between institutions and students. At the institution-to-institution level, the local institution serves as the mediator bridging the foreign educational resources and the local contexts, and the foreign institution adapts to local conditions with trust and restrained power. At the institution-to-student level, developing a share-culture in management and quality assurance is significance for the sustainability of TNE, since the Western institutions take an egalitarian student-centered approach while the Southeast Asian countries prefer hierarchical structures embracing control over students and the actions of faculty (Mirkasimov et al., 2021). In this case, both institutions adopt the egalitarian student-centered approach in programming, which can be found in the process of classroom teaching, assessment and other service management activities. That sympathetic lecturers were perceived as the significant factors to students’ success presents the evidence of an equal dialogue between institutions and students.

## Conclusion

In summary, the analysis of semi-structure interviews with seven participants and documentations of program handbook and module assessment form presented two important themes indicating the factors influencing the service quality, i.e., the high-quality educational resources that satisfy student personal growth and host country’s social development, and the professional and sympathetic lecturers that offer support to student successes. Further discussions were made to reveal the logic for sustainability of TNE programs.

Practical implications from the current study can be made to different stakeholders in TNE. First and the most, TNE should be confirmed as one kind of service for program management. This will offer a service quality perspective to quality assurance in TNE, in which student satisfaction should be considered as an important indicator of service quality, meanwhile the learning outcomes should be emphasized in service quality evaluation. Second, local institutions should insist on the self-centered ideology in cooperation and programming. Local institutions are the gate keeper of introducing high-quality educational resources, and the mediators bridging the local needs and the foreign resources. Local institutions should assume the main responsibility of TNE quality. Third, foreign institutions must insist on exporting high-quality educational resources to the host country and adapt to the host countries’ conditions. The automatic inbuilt power in academic criteria, assessment and other decision-making from the degree-awarded institutions should be rationally used.

## Limitations and future research

The major limitation is my positioning as the program administrative staff and researcher in this study, which would typically lead to biased problems due to my own experiences and misunderstandings. The first suggestion for future studies is to replicate this student-centered qualitative evaluation study on other TNE programs to provide more in-depth understanding of factors influencing the service quality, such as the programs on different levels and disciplines. The second direction for future research would be to conduct a comparative study on the local programs and TNE programs run in a same host institution, since they share similar tangibles and lecturers and hopefully will provide understandings of inter-cultural factors.

## Data availability statement

The raw data supporting the conclusions of this article will be made available by the authors, without undue reservation.

## Ethics statement

The studies involving human participants were reviewed and approved by Faculty of English Language and Culture, Guangdong University of Foreign Studies. The patients/participants provided their written informed consent to participate in this study.

## Author contributions

HO contributed to design of the study and audit of data analysis. CZ conducted the data collection, data analysis and writing up of the findings. All authors contributed to the article and approved the submitted version.

## Funding

This work was supported by a Humanities and Social Sciences Planning Foundation of Ministry of Education of China (no. 20YJA880040), and a Guangdong Province Philosophy and Social Sciences Planning Project (no. GD22WZX02–08).

## Conflict of interest

The authors declare that the research was conducted in the absence of any commercial or financial relationships that could be construed as a potential conflict of interest.

## Publisher’s note

All claims expressed in this article are solely those of the authors and do not necessarily represent those of their affiliated organizations, or those of the publisher, the editors and the reviewers. Any product that may be evaluated in this article, or claim that may be made by its manufacturer, is not guaranteed or endorsed by the publisher.
